# The Danger of Typhus

**Published:** 1920-09-25

**Authors:** 


### THE DANGER OF TYPHUS.

The Imperial War Relief Fund which has been established to co-ordinate all existing British agencies for the relief of European distress—and forty Societies are estimated to be engaged in it—and to fight the typhus epidemic which threatens to overrun the whole continent, is trying to arouse attention to the extent of the danger. From the Baltic to the Black Sea there is generally an absolute lack of medicines and sanitary appliances; even soap and disinfectants cannot be obtained; and doctors, nurses, and hospital equipment are lacking. In Poland alone 231,206 cases of typhus were officially notified last year, and this number is believed to be but half of the gross total. In Soviet Russia 1,600,000 cases were notified last year, and the war between Poland and Russia has broken the barrier between the two countries, and threatens to disseminate infected refugees in all directions towards the West. With the coming of winter, a season congenial to the disease, the prospect is immensely serious, and at present there is no effective machinery to meet it, unless the Imperial War Relief Fund can raise the three and a quarter millions for which it asks. The League of Red Cross Societies, of which Sir David Henderson is Director-General, will unify effort if the necessary means are found.

The Conference on International Health, held in London last April, presented a plan of campaign to the Council of the League of Nations to arrest the spread of typhus in Poland, which is regarded as the strategic point. The plans of the Polish authorities were approved, but must be reinforced and much extended by the establishment of two dozen more quarantine stations and 30,000 hospital beds. To secure a general cleansing of the population 500 mobile units are also required; sheets, blankets, and cotton shirts are needed by the hundred thousand; 100 ambulances; 2,000 tons of soap. To staff the first five quarantine stations and ten 100-bed hospitals, 65 doctors, 150 nurses, and 10 sanitary inspectors are required. The work will not be limited to Poland, but extended to other countries, including Russia, as opportunity and need occurs.

### An Opportunity.

There has been much talk of late of the virtue of preventive medicine. We have now an opportunity to practise it. Unfortunately, people are apt to measure dangers by the noise they make; and, as Mr. A. J. Balfour lately said, the British public is in almost complete ignorance of the condition of Central Europe.

